# Supplementary material for: Multiplexed storage and real-time manipulation based on a multiple degree-of-freedom quantum memory
Source: Nat Commun. 2018 Aug 24;9:3407. doi: 10.1038/s41467-018-05669-5 (PMC6109076; doi:10.1038/s41467-018-05669-5)
Supplement: Supplementary file 1 — Supplementary Information [file 41467_2018_5669_MOESM1_ESM.pdf]

# Supplementary information: Multiplexed storage and real-time manipulation based on a multiple-degree-of-freedom quantum memory

### Supplementary Note 1 - Atomic frequency comb structure and memory efficiency

The method used to prepare the atomic frequency comb (AFC) is described in the main paper. An example of a single AFC structure in the memory crystal (MC) is shown in Supplementary Figure 1 (black line). The AFC has a peak linewidth of $\gamma$ = (81.11 ± 2.40) kHz and a periodicity of $\Delta$ = 200 kHz. The comb has an absorption depth of $d = 3.38 \pm 0.20$ and a background optical depth of $d_0 = 0.28 \pm 0.05$. The finesse is $F = \Delta/\gamma = 2.47 \pm 0.03$. The predicted efficiency [1] for Gaussian AFC peaks is $\eta_{AFC} = (d/F)^2 \mathrm{e}^{-d/F} \mathrm{e}^{-7/F^2} \mathrm{e}^{-d_0}$, corresponding to $\eta_{AFC,th}$ = 11.37%. For comparison, the typical measured value of the AFC efficiency is $\eta_{AFC,\ exp}$ = 11.07%. The spin-wave storage efficiency [2] is $\eta_{SW} = \eta_{AFC} \times \eta_T^2 \times \eta_C$. We measured $\eta_{SW}$ = 5.51%, from which we can deduce the transfer efficiency of control pulse $\eta_T$ = 81.5% (under the assumption of efficiency caused by spin decoherence $\eta_C$∼75% [2]). We prepared the narrow-band spectral filter [3] in the filter crystal (FC) simultaneously with the AFC. The frequency was scanned over 0.8 MHz around the input frequency to create a transparency window. As shown in Supplementary Figure 1 (red line), the transparency window has a width of FWHM = 1.84 MHz, which approximately matches the AFC bandwidth of 2 MHz. The second SLM has a diffraction efficiency of 60%. The diffraction efficiency of the AOM gates is approximately 41%. The diffracted signal from the AOM gates is coupled into a SMF (efficiency: 85%) and sent to the FC. The double-passed FC has a total transmission of approximately 38%. The final signal is coupled into a SMF (efficiency: 75%) and detected by the SPD. The total transmission of the input beam from the memory crystal to the SPD is approximately 5.9%. The SPD has a detection efficiency of approximately 60%.

We created two AFC in the MC with an interval of 80 MHz between them to achieve spectral multiplexing. In order to achieve the temporal multiplexing, the duration of input pulses needs to be reduced. The duration of the input pulses is reduced from 500ns to 390ns, which resulted into a slight reduction of memory efficiency due to the larger bandwidth of input pulses. In this case, the AFC efficiencies are $\eta_{AFC,\ exp}$ = 10.78% and $\eta_{AFC,\ exp}$ = 10.55%, for the first AFC and the second AFC, respectively. The spin-wave storage efficiencies are $\eta_{SW}$ = 5.05% and $\eta_{SW}$ = 5.13%, for the first AFC and the second AFC, respectively.

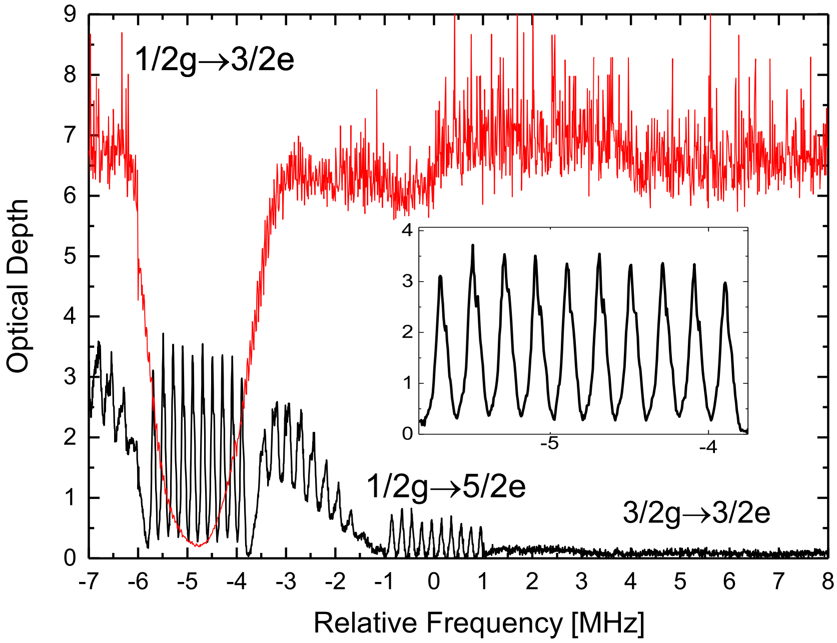


**Supplementary Figure 1: Single atomic frequency comb structure.** The black line represents the AFC structure in the MC, and the red line represents the filter structure in the FC.

### Supplementary Note 2 - Storage for two dimensional orbital angular momentum states.

We present more detailed measurements of superposition states of the form $(|\mathrm{L}\rangle + |\mathrm{R}\rangle)/\sqrt{2}$. The pattern on the first spatial light modulator (SLM) was programmed to prepare the required superposition state $(|\mathrm{L}\rangle + |\mathrm{R}\rangle)/\sqrt{2}$, whereas the measurement basis, as controlled by SLM2, was set to $(|\mathrm{L}\rangle + \mathrm{e}^{i\theta - i\pi/2}|\mathrm{R}\rangle)/\sqrt{2}$, with $\theta$ varying from 0 to 360 degrees. In Supplementary Figure 2, the black dots represent the measured results, and the red line represents a sinusoidal fit to the data. For $\mu = 1.18$, the measured visibility is 93.6% $\pm$ 2.9%. The visibility achieved here is slightly greater than the fidelity of the memory process in all three dimensions. This is because the efficiencies for the Gaussian and LG modes are not balanced. Further increasing the beam diameter or using a super-Gaussian spatial profile for the pump/control light could improve the higher-dimensional memory performance.

### Supplementary Note 3 - Quantum state tomography of orbital angular momentum qutrits.

To benchmark the performance for quantum mode conversion and arbitrary manipulations, we performed quantum state tomography [4] for orbital-angular-momentum (OAM) qutrits to assess the state fidelity after these operations. We first measured the input states before the quantum memory and reconstructed the density matrices of $|\psi_1\rangle$ and $|\psi_2\rangle$.The fidelity of the prepared $|\psi_1\rangle$ and $|\psi_2\rangle$ are 0.903 $\pm$ 0.004 and 0.895$\pm$ 0.008 with respect to the ideal states respectively. As typical examples, Supplementary Figure 3 presents graphical representations of the reconstructed density matrices for the OAM qutrit states $|\psi_1\rangle$ and $|\psi_2\rangle$ after mode conversion from $f_2t_2$ to $f_1t_1$ and from $f_1t_1$ to $f_2t_2$. The $|\mathrm{G}\rangle\langle\mathrm{G}|$ component is higher than all the others as shown in Supplementary Figure 3. This is because of the imperfection of the setup and the action of the memory. Due to the imperfection in preparation and detection, the $|\mathrm{G}\rangle\langle\mathrm{G}|$ component is higher than other LG modes before memory. The storage process will further increase this deference due to the limited diameter of pump/control beam.

### Supplementary Note 4 - Theoretical model of fidelity.

At first, we characterized the memory fidelity involving bright coherent pulses ($\mu \gg 1$) with negligible noise caused by the storage process. The classical bound for the memory fidelity can be derived from the visibility of output with a classical input signal [5, 6]:

$$F_C = (1 + V_C)/2 = S_{max}/(S_{max} + S_{min}) \tag{1}$$

We prepared bright coherent pulses in orthogonality states and achieved a mean classical fidelity of 99.0%. If the memory is implemented with true single photon source, the classical fidelity bound is 2/3 [7]. We then measured the unconditional noise floor $p_n$ and memory efficiency $\eta$ for our device. Then, we can obtain the formula for the fidelity when the input contains $\mu$ photons [5, 6]:

$$F_q = \frac{F_C + \mu_1/\mu}{1 + 2\mu_1/\mu} \tag{2}$$

Where $\mu_1 = p_n/\eta$ is a new parameter.

Since weak coherent pulses are employed as the input for experiment, the number of photons in a pulse mu is assumed to follow the Poisson distribution $P(\mu, n) = e^{-\mu}\frac{\mu^n}{n!}$ The conditional fidelity for memory output can be calculated as [5, 6]:

$$F_M(\eta) = \sum_{n=1} \frac{n+1}{n+2}\frac{P(\mu, n)}{1 - P(\mu, 0)} \tag{3}$$

Considering the non ideal memory efficiency, the conditional fidelity can be written as [5, 6]:

$$F_M(\mu, \eta_M) = \frac{\frac{n_{min}+1}{n_{min}+2}\gamma + \sum_{n\geq n_{min}} \frac{n+1}{n+2}P(\mu, n)}{\gamma + \sum_{n\geq n_{min}} P(\mu, n)} \tag{4}$$

where $0 < \gamma < P(\mu, n_{min})$ and $n_{min} = min\ i : \sum_{n\geq i+1} P(\mu, n) \leq (1 - P(\mu, 0))\eta_M$. This is the maximal fidelity achievable using a classical memory device which already takes into account the finite memory efficiency and the Poissonian statistics of the weak coherent pulses [2, 8]. The different lines corresponding to the above discussed cases

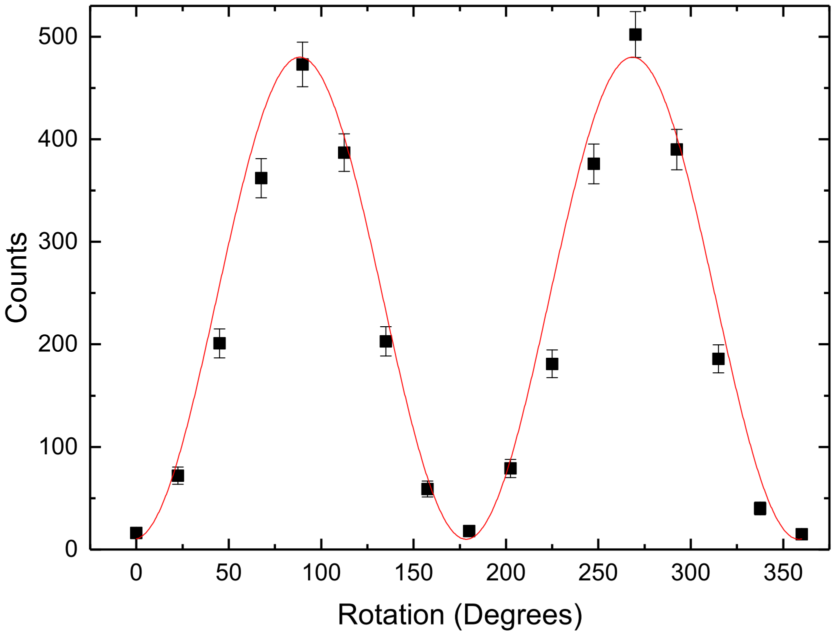


**Supplementary Figure 2: Detailed measurements of the superposition state $(|\mathbf{L}\rangle + |\mathbf{R}\rangle)/\sqrt{2}$.** The black dots represent the measured results, and the red line represents a sinusoidal fit to the data. The error bars in these data are due to the photon counting statistics.

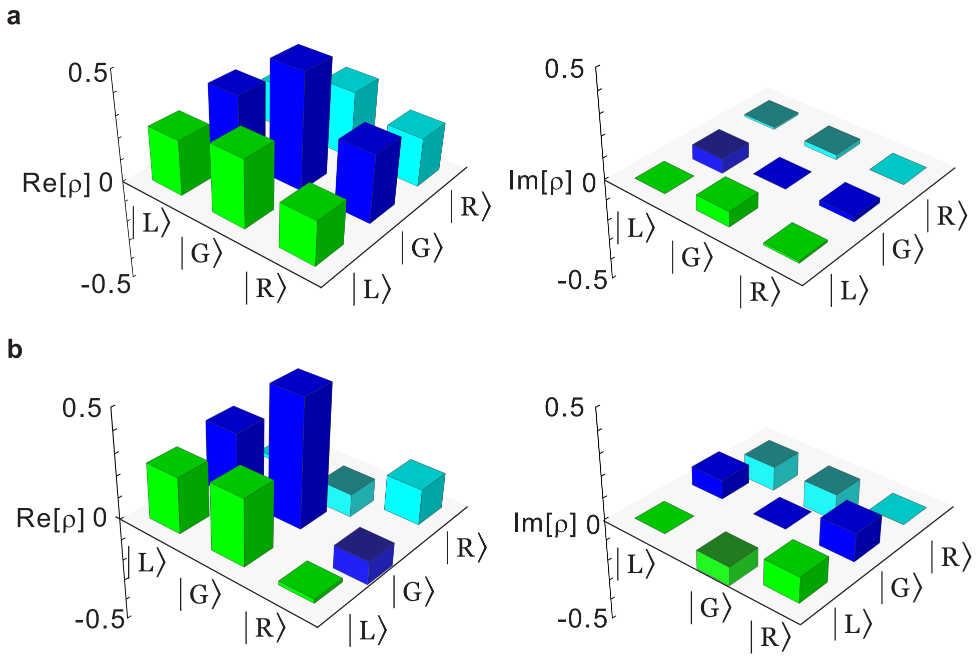


**Supplementary Figure 3: Quantum state tomography of mode converted orbital angular momentum qutrits.** **a**, Reconstructed density matrix for the OAM qutrit state $(|\mathrm{L}\rangle + |\mathrm{G}\rangle + |\mathrm{R}\rangle)/\sqrt{3}$ after mode conversion from $f_2t_2$ to $f_1t_1$. **b**, Reconstructed density matrix for the OAM qutrit state $(|\mathrm{L}\rangle + |\mathrm{G}\rangle$ - $\mathrm{i}|\mathrm{R}\rangle)/\sqrt{3}$ after mode conversion from $f_1t_1$ to $f_2t_2$.

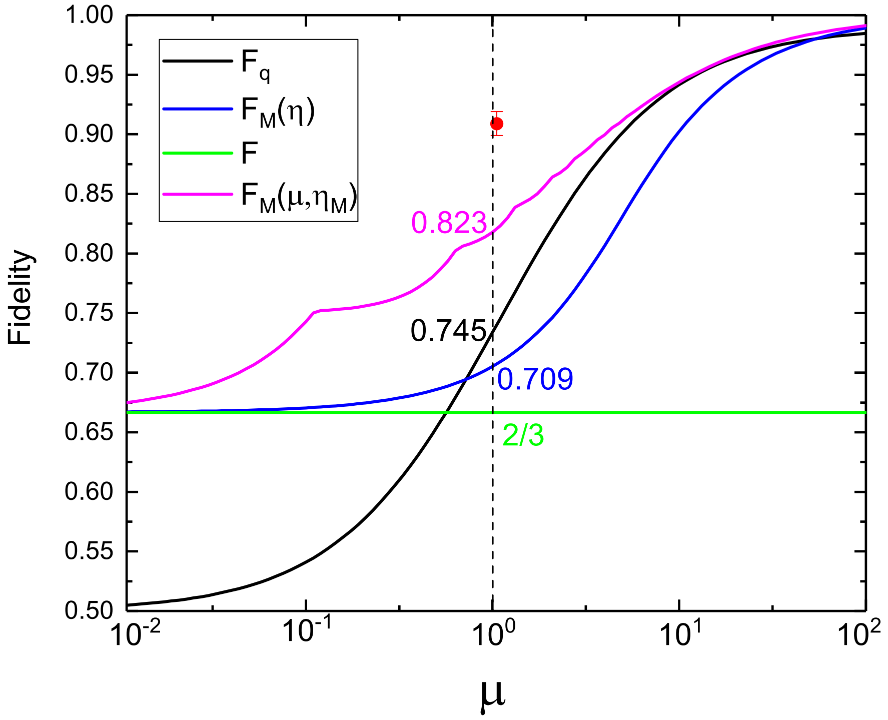


**Supplementary Figure 4: Classical bound of memory fidelity vs input photon number per pulse.** The green line is the fidelity limit for storage of true single photon using a classical memory. The black line corresponds to Supplementary Equation 2 for $\mu_1 = 0.5$. The blue line corresponds to Supplementary Equation 3 where only the photon number distribution is taken into account. The pink line corresponds to Supplementary Equation 4 where the photon number distribution and the storage efficiency are taken into account. The vertical black dashed line is when the $\mu = 1$. The measured fidelity of storage process for three dimensional OAM states is indicated using a red dot.

are plotted in Supplementary Figure 4 as a function of $\mu$. For our experimental condition ($\mu \sim 1$, $\eta_M \sim 5.1\%$), the classical bound of fidelity is 0.823, as calculated based on Supplementary Equation 4.

---

**Supplementary References**


[1] Afzelius, M., Simon, C., de Riedmatten, H. & Gisin, N. Multimode quantum memory based on atomic frequency combs. *Phys. Rev. A* **79**, 052329 (2009).

[2] Gündoğan, M., Ledingham, P. M., Kutluer, K., Mazzera, M. & de Riedmatten, H. Solid state spin-wave quantum memory for time-bin qubits. *Phys. Rev. Lett.* **114**, 230501 (2015).

[3] Beavan, S. *et al.* Demonstration of a dynamic bandpass frequency filter in a rare-earth ion-doped crystal. *J. Opt. Soc. Am. B* **66**, 012303 (2002).

[4] Thew, R. T., Nemoto, K., White, A. G. & Munro, W. J. Qudit quantum-state tomography. *Phys. Rev. A* **30**, 1173 (2013).

[5] Laplane, C. *et al.* Multiplexed on-demand storage of polarization qubits in a crystal. *New J. Phys.* **18**, 013006 (2015).

[6] Gündoğan, M. *et al.* Quantum storage of a photonic polarization qubit in a solid. *Phys. Rev. Lett.* **108**, 190504 (2012).

[7] Specht, H. P. *et al.* A single-atom quantum memory. *Nature.* **473**, 190-193 (2011).

[8] Vernaz-Gris, P. *et al.* Highly-efficient quantum memory for polarization qubits in a spatially-multiplexed cold atomic ensemble. *Nat. Commun.* **9**, 363, (2018).
